# Adverse Mentions, Negative Sentiment, and Emotions in COVID-19 Vaccine Tweets and Their Association with Vaccination Uptake: Global Comparison of 192 Countries

**DOI:** 10.3390/vaccines10050735

**Published:** 2022-05-08

**Authors:** Jungmi Jun, Ali Zain, Yingying Chen, Sei-Hill Kim

**Affiliations:** School of Journalism and Mass Communications, College of Information and Communications, University of South Carolina, Columbia, SC 29208, USA; junj@mailbox.sc.edu (J.J.); yychen@sc.edu (Y.C.); kim96@mailbox.sc.edu (S.-H.K.)

**Keywords:** COVID-19, infodemic, vaccine, adverse events, side-effect, sentiment, emotions, social media, Twitter, artificial intelligence (AI), country, global, multinational

## Abstract

*Background*: Many countries show low COVID-19 vaccination rates despite high levels of readiness and delivery of vaccines. The public’s misperceptions, hesitancy, and negative emotions toward vaccines are psychological factors discouraging vaccination. At the individual level, studies have revealed negative perceptual/behavioral outcomes of COVID-19 information exposure via social media where misinformation and vaccine fear flood. *Objective*: This study extends research context to the global level and investigates social media discourse on the COVID-19 vaccine and its association with vaccination rates of 192 countries in the world. *Methods*: COVID-19 vaccine tweets were compared by country in terms of (1) the number per million Twitter users, (2) mentions of adverse events—death, side-effects, blood clots, (3) negative sentiment (vs. positive), and (4) fear, sadness, or anger emotions (vs. joy). Artificial intelligence (AI) was adopted to classify sentiment and emotions. Such tweets and covariates (COVID-19 morbidity and mortality rates, GDP, population size and density, literacy rate, democracy index, institutional quality, human development index) were tested as predictors of vaccination rates in countries. *Results*: Over 21.3 million COVID-19 vaccine tweets posted between November 2020 and August 2021 worldwide were included in our analysis. The global average of COVID-19 vaccine tweets mentioning adverse events was 2% for ‘death’, 1.15% for ‘side-effects’, and 0.80% for ‘blood clots’. Negative sentiment appeared 1.90 times more frequently than positive sentiment. Fear, anger, or sadness appeared 0.70 times less frequently than joy. The mention of ‘side-effects’ and fear/sadness/anger emotions appeared as significant predictors of vaccination rates, along with the human development index. *Conclusions*: Our findings indicate that global efforts to combat misinformation, address negative emotions, and promote positive languages surrounding COVID-19 vaccination on social media may help increase global vaccination uptakes.

## 1. Introduction

Multiple types of vaccines for a newly discovered coronavirus, SARS-CoV-2 (COVID-19 hereafter), have become available globally, although the level of public accessibility varies by nation. There are 33 vaccines approved by at least one country, and the World Health Organization (WHO) has approved 10 types to date (Nuvaxovid, COVOVAX, Moderna/Spikevax, Pfizer/BioNTech, Janssen, Oxford/AstraZeneca, Covishield, Covaxin, Sinopharm/Covilo, and Sinovac/CoronaVac) [1]. All 195 countries that are members or observers of the United Nations have approved at least one vaccine. As of March 2022, 63.5% of the world population has received at least one dose of a COVID-19 vaccine [2], remaining below the goal of WHO to reach herd immunity [3]. Global inequity of COVID-19 vaccines exists: only 13.7% of people in low-income countries have received at least one dose of vaccine [2]. Fewer than 15 countries (e.g., Canada, United States, Italy, United Kingdom, Germany) have enough vaccines to cover the entire population, and many countries rely on the import or donation of vaccines from others [4]. However, there are sufficient vaccine supplies/leftovers for most of the world [5]. 

The percentage of the fully vaccinated population remains below 70% in many developed countries, including the United States, despite ample vaccine supplies and more than 1 year of inoculation efforts [6]. The low vaccination rate in these countries seems to be an outcome of individuals’ choice not to get vaccinated, not due to supply or access issues. Such an individual decision can be associated with misperceptions, hesitancy, and negative emotions toward vaccines [7]. 

Social media has been a critical source of COVID-19 information [8]. However, social media has been flooded with misinformation and disinformation since the beginning of the pandemic [9]. These platforms are also where people express and circulate individual opinions and emotions—either negative or positive—toward vaccination. Research found that COVID-19 vaccine-related tweets were emotional, and many showed objection and hesitancy rather than interest in vaccines [10]. 

At the individual level, research has evidenced the negative impacts of social media on COVID-19-related perceptions and behaviors. Individuals who were exposed to COVID-19 information via social media were less likely to have optimistic attitudes and adhere to preventive measures as compared to those who used authoritative sources (i.e., the Ministry of Health) [11]. Those who trusted COVID-19 news from social media were more likely to believe in COVID-19 myths and false information, while those who trusted information from the government were less likely to do so [12]. COVID-19 misinformation exposure via social media or instant messaging was also associated with lower engagement in preventive behaviors, mediated by misinformation beliefs [13]. 

On the basis of the literature, we propose that a country’s COVID-19 vaccination discourse on social media will be an important predictor of the country’s vaccination rate. A higher presence of negative sentiment and emotions on social media discourse of vaccination in a country will be associated with the public’s tendency of negative perceptions and attitudes toward vaccination and, thus, will be associated with a lower vaccination rate of the country. Multiple social science theories supporting the influence of languages, opinions/climate, and/or social networks on social media on health behaviors of a group of people exist: social cognitive theory [14], media framing theory [15], and social contagion theory [16]. Extant studies have identified the type, source, diffusion, and individual impact of COVID-19 (mis)information on social media within a country [13,17,18]. Yet, few studies have utilized a multinational approach comparing vaccine discourse on social media across countries. Moreover, it is unknown if a country’s social media conversations on COVID-19 vaccination are associated with actual vaccination rate of the country. Given the context and the gap in the literature, this study compares countries’ COVID-19 vaccination discourse on Twitter in terms of the mentions of adverse events, sentiment, and emotions, as well as tests such factors as predictors of vaccination rates among countries. 

**Death in COVID-19 vaccine tweets.** Early news reporting and social media discourses surrounding COVID-19 vaccines focused on their safety and potential adverse events, such as death and side-effects. Research found that deaths and side-effects were two of the most frequently appeared topics in COVID-19 vaccine-related tweets posted between November 2020 and February 2021 [19]. More than 100 deaths after vaccination were reported to the US government’s Vaccine Adverse Event Reporting System (VAERS; where anyone, including non-health experts, can submit suspected events) in the first month of inoculation [20]. Although the US Centers for Disease Control and Prevention (CDC) reviewed and reported that these deaths were not related to COVID-19 vaccination, numerous posts misinforming or misinterpreting VAERS reports or including rumors on a death after vaccine spread on social media [21]. The experiences of the US as one of the first countries to approve COVID-19 vaccines and begin inoculation were likely to be shared globally via social media. For instance, Facebook announced that a healthy doctor dying 2 weeks after taking COVID-19 vaccine was the most viewed content on its platform in the first 3 months of 2021 [22].

**Blood clots in COVID-19 vaccine tweets.** In December 2020, the US Food and Drug Administration (FDA) and CDC determined to pause the use of the Johnson and Johnson COVID-19 vaccine in the US due to reports of a rare and severe type of blood clot, and the agencies lifted the order and recommended to resume of the use of the vaccine after a safety review in April 2021 [23]. Furthermore, scientists found a potential link between blood clots and AstraZeneca [24], another COVID-19 vaccine approved and widely used in the United Kingdom and other countries [1]. The scientists explained the type of blood clots induced by the vaccine to be extremely rare, linked to 73 deaths out of nearly 50 million doses of AstraZeneca given in the UK [25]. Accordingly, blood clots, as a representative side-effect of COVID-19 vaccine, have received tremendous attention from news media and the global public [26]. 

**Negative sentiment in COVID-19 vaccine tweets.** The global public has shown different valence and attitudes toward COVID-19 vaccination. Studies based on sentiment analysis of tweets revealed that users often posted words indicating negative, positive, or neutral sentiment to express attitudes toward COVID-19 and health-related issues [19,27,28]. On social media, users expressed negative attitudes related to COVID-19 vaccination using words indicating negative sentiment. For example, a recent study analyzed 2.6 million tweets about COVID-19 vaccines in the English language and found that tweets with negative sentiment were more likely to mention conspiracy theories or question the trial results or efficacy of COVID-19 vaccines than their counterparts (i.e., tweets with positive or neutral sentiment) [29]. Thus, understanding the proportion of negative sentiment as compared to positive sentiment in COVID-19 vaccine tweets from a country can help quantify vaccine-related opinions of the country. 

**Fear, sadness, and anger in COVID-19 vaccine tweets.** Research suggests that emotions are often stronger predictors of vaccine risk perceptions and intentions than statistical risk [30]. In the COVID-19 vaccine context, fear and anxiety were significantly associated with vaccine acceptance [31]. Examining the specific emotions expressed in tweets can help quantifying widespread emotions associated with COVID-19 vaccination in a country. Recent studies found the dominance of fear emotions when people tweet about new COVID-19 cases and deaths [32], as well as continuous diffusions of fear, anxiety, stress, and depression in COVID-19-related tweets [33]. 

### Objectives

The current study aimed to analyze COVID-19 vaccine tweets and test their association with the vaccination rates of 192 countries in the world. We compared COVID-19 vaccine tweets by country in terms of (1) the number of related tweets per million Twitter users, (2) the proportion of tweets mentioning adverse events (death, side-effects, blood clots), (3) appearance of negative sentiment as compared to positive sentiment as classified by an AI tool, and (4) appearance of fear, sadness, or anger emotions as compared to joy as classified by an AI tool. To address the last aim, we tested the adverse mentions, negative sentiment, and fear/sadness/anger emotions in tweets, along with the COVID-19 morbidity/mortality and socioeconomic and political characteristics of a country, as predictors of its vaccination rate.

## 2. Method

### 2.1. Data Collection

To collect our data (COVID-19 vaccine-related tweets from countries in the world), Brandwatch (social media/online data search and analytics tool) was employed. The search location included 192 countries that are members/observers of the United Nations [34] and have access to the Twitter platform [35]. Two groups of queries were used: (1) “COVID-19” or “coronavirus” and (2) “vaccine” or “vaccination”. We used the keywords in English, as well as those translated in the dominant languages of each country if the languages were supported by Twitter. A total of 33 languages (English, Arabic, Bengali, Czech, Danish, German, Greek, Spanish, Persian, Finnish, Filipino, French, Hebrew, Hindi, Hungarian, Indonesian, Italian, Japanese, Korean, Malay, Dutch, Norwegian, Polish, Portuguese, Romanian, Russian, Swedish, Thai, Turkish, Ukrainian, Urdu, Vietnamese, Chinese (simplified), and Chinese (traditional)) were used in our search. The search period was from 1 November 2020 (the beginning of vaccine approval and global inoculation efforts) to 15 August 2021 (the data collection date). Noise in tweets was excluded (e.g., advertisements, pornography, and automatic tweet generation apps) using a filter in Brandwatch.

### 2.2. Measures

**COVID-19 vaccine tweets per million Twitter users.** We considered the population size and Twitter penetration rate in calculating the number of COVID-19 vaccine tweets of each country to compare the degree of attention to the topic of COVID-19 vaccine on Twitter by country. Twitter penetration rate varied by country from 0.03% in Eritrea to 52.6% in Luxembourg [36]. We divided the total number of COVID-19 vaccine tweets of a country by million population and Twitter penetration rate. For example, we found a total of 6,570,155 tweets mentioning COVID-19 vaccines in the United States, which has a population of 332,915,074 and a Twitter penetration rate of 27.7%. In our analysis, the COVID-19 vaccine tweets per million Twitter users for the United States was calculated as 5466.66 = 6,570,155 ÷ 332.9 × 0.27.

**Adverse mentions—“death”, “side-effects”, and “blood clot”**. We calculated the proportion of COVID-19 vaccine tweets mentioning words signifying adverse events of vaccination, including *death* (“death” or “die”), *side-effects* (“side-effect” or “side-effects”), and *blood clot* (“blood clot” or “blood clots”). Again, these mentions were searched in English and an additional 33 languages. 

**Negative sentiment.** Brandwatch’s sentiment analysis tool was used to measure the sentiment. Sentiment analysis is a natural language processing (NLP) technique that categorizes documents into negative-, positive-, and neutral-based classification algorithms [37]. According to Brandwatch, its sentiment analysis tool is based on transfer learning, which is a deep learning method, reaching an average accuracy rate of 60–75% across 44 languages [38]. The transfer learning approach improves the accuracy of results by supervised machine learning models or a dictionary-based approach [39]. We should note that the sentiment analysis does not reveal the ground truth; therefore, we set positive sentiment as a benchmark to examine the relative changes of negative sentiment. We adopted the negative and positive sentiment estimated and classified by the sentiment analysis tool and calculated the odds of negative sentiment as compared to positive sentiment. 

**Fear, sadness, or anger emotions.** We relied on Brandwatch’s emotion analysis tool to identify tweets that express fear, sadness, or anger emotions. The statistical classification models categorized each tweet into one of six emotions—anger, disgust, fear, joy, surprise, and sadness [40]. Like sentiment analysis, the emotion analysis tool does not reveal the ground truth. Therefore, we set joy as the benchmark emotion and calculated the odds of fear, anger, or sadness as compared to joy. 

**Vaccination rate of a country.** The outcome variable, vaccination rate of each country, was operationalized as the percentage of total adult population that received at least one dose of any vaccine as of December 2021, which was obtained from Our World in Data [6], World Bank [41], European Centre for Disease Prevention and Control [42], and the US Centers for Disease Control and Prevention [43].

**Covariates:** We considered *COVID-19 morbidity* (COVID-19 infection cases per million population) and *mortality* rates (COVID-19 linked deaths per million population) as of December 2021 for each country, adopted from the Economist Intelligence Unit [44]. In addition, we included socioeconomic and political determinants that are known to predict the access to vaccines or actual vaccination rate of nations [45]: *total population, population density,* and *GDP per capita* adopted from Mathieu et al. [6], *literacy* adopted from Roser and Ortiz-Ospina [46], *democracy index* (each country’s scores for electoral process and pluralism, functioning of government, political participation, democratic political culture and civil liberties: 1 = authoritarian regime, 2 = hybrid regime, 3 = flawed democracy, 4 = full democracy) adopted from the Economist Intelligence Unit [44], and *institutional quality* (voice and accountability, political stability and absence of violence/terrorism, government effectiveness, regulatory quality, rule of law, and control of corruption indices; lowest score = −3.00, highest score = 3.00), and *human development index* (composite index of life expectancy, education, and per capita income indicators, which are used to rank countries into four tiers of human development: 1.0–0.8 = very high, 0.79–0.70 = high, 0.70–0.55 = medium, below 0.55 = low) adopted from Kaufmann and Kraay [47].

**Figure A1:**
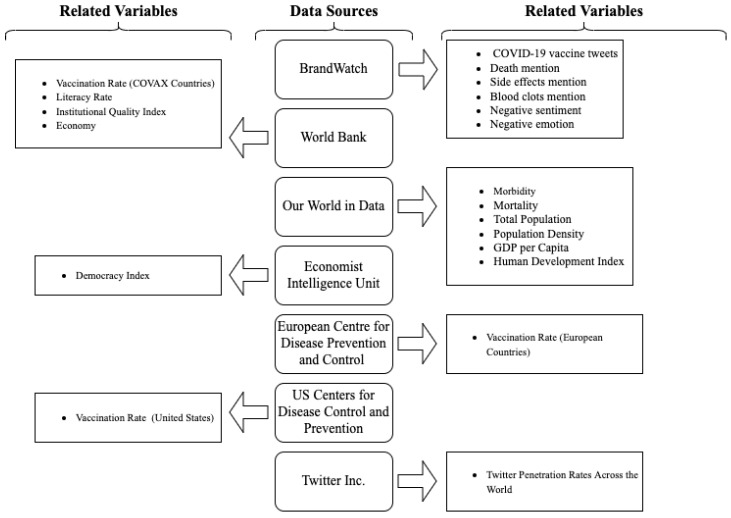
Study variables and data sources.

All variable used and the sources of secondary data are summarized in Figure A1. Study variables and data sources. 

## 3. Results

Table 1 presents the descriptive information of covariates used in this study, including the means, dispersion, and ranges of COVID-19 morbidity and mortality, vaccination rate (as of December 2021), and socioeconomic and political characteristics of 192 counties included in our analysis. 

Table 2 shows the global average of COVID-19 vaccine tweet-related variables and top 10 countries with higher rates or odds. There were over 21.3 million COVID-19 vaccine tweets worldwide and nearly 2740 COVID-19 vaccine tweets per million people in the world. We found about 350 COVID-19 vaccine tweets per million Twitter users on average, posted between November 2020 and August 2021. Countries posting greater numbers of COVID-19 vaccine tweets per million users include Nigeria (*N* = 2586) followed by Tonga (*N* = 2510), St. Vincent and the Grenadines (*N* = 2274), and Fiji (*N* = 2160).

Of the COVID-19 vaccine tweets, nearly 2% mentioned ‘death’ on a global average. Countries showing a higher proportion of ‘death’ mentions included Germany (25.60%), Austria (21.60%), Japan (12.90%), the Netherlands (10.68%), and Liechtenstein (10.64%). As for ‘side-effects’, 1.15% of COVID-19 vaccine tweets mentioned such words, with Burundi (4.16%), Comoros (4.00%), and Germany (3.47%) mentioning ‘side-effects’ more frequently. About 0.80% of COVID-19 vaccine tweets mentioned ‘blood clots’ globally. 

Our sentiment analysis showed that negative sentiment was more likely to appear than positive sentiment in COVID-19 vaccine tweets globally (M = 1.90 times, SD = 1.33). The likelihood of negative sentiment (vs. positive) was greater in some countries, including Turkey (11.93 times), Burundi (8.73 times), Japan (6.79 times), Congo (6.68 times), and Burma (5.18 times).

The emotion analysis showed that, globally, fear, sadness, or anger appeared less frequently than joy in COVID-19 vaccine tweets (M = 0.70 times, SD = 0.33). One of the three negative emotions appeared more frequently than joy in some countries including Namibia (1.87 times), Australia (1.65 times), Eritrea (1.63 times), Burma (1.60 times), and South Africa (1.55 times). 

The 192 countries included in our analysis and their number of COVID19 vaccine tweets, adverse mentions, sentiment, and emotions are available in Table S1. 

Table 3 entails the results of Pearson’s correlation analyses. Notable findings were significant and positive correlations between ‘death’ and ‘side-effect’ mentions (*r* = 0.414, *p* < 0.001), between ‘side-effect’ mentions and negative sentiment (*r* = 0.338, *p* < 0.001), and between ‘blood clot’ mentions and fear/sadness/anger emotions (*r* = 0.316, *p* < 0.001). COVID-19 morbidity rates showed a positive correlation with all three adverse mentions: ‘death’ (*r* = 0.186, *p* < 0.001), ‘side-effects’ (*r* = 0.224, *p* = 0.002), and ‘blood clots’ (*r* = 0.257, *p* < 0.001).

Overall, country’s GDP per capita, democracy index, institutional quality, and human development index were positively associated with adverse mentions and fear/sadness/anger emotions in COVID-19 vaccine tweets. 

Table 4 shows the results of Pearson’s correlation and hierarchical regression analyses, which were used to determine predictors of vaccination rates of countries. A three-step hierarchical regression analysis was performed. In all models, COVID-19 morbidity and mortality were entered in the first block. Socioeconomic and political characteristics of countries, including GDP per capita, total population, population density, literacy rate, democracy index, institutional quality, and human development index (HDI), were entered in the second block. COVID-19 vaccine tweet factors including the number of COVID-19 vaccine tweets per million users, the proportion of adverse mentions (death, side-effects, and blood clot), negative sentiment, and fear/sadness/anger emotions were entered in the last block. 

COVID-19 morbidity significantly predicted vaccination rates (β = 0.538, SE = 0.000, *p* < 0.001; see Model 1 column in Table 4), while COVID-19 mortality did not. However, the effect of COVID-19 morbidity did not remain when socioeconomic and political characteristics of country were considered. As shown in the Model 2 column, human development index (β = 0.734, SE = 24.490, *p* < 0.001) appeared as the strongest predictor. In the final model including all covariates, ‘side-effect’ mentions (β = −0.156, SE = 1.889, *p* < 0.01) and fear/sadness/anger emotions (β = −0.105, SE = 4.630, *p* < 0.05) appeared as significant predictors of vaccination rates, along with human development index (β = 0.682, SE = 25.275, *p* < 0.001). In the final model, A significant regression equation was found for COVID-19 morbidity and mortality in the first block (F [2, 151] = 23.85, *p* < 0.001), with an *R*^2^ of 0.24, socioeconomic and political characteristics of country in the second block (F [7, 144] = 36.02, *p* < 0.001), with an *R*^2^ of 0.48, and COVID-19 vaccine tweet factors in the last block (F [6, 138] = 4.90, *p* < 0.001), with an *R*^2^ of 0.05. The total *R*^2^ of the regression model was 0.77.

## 4. Discussion

The WHO declared an *infodemic* in referring to the wide spread of false and misleading information about COVID-19 in digital and physical environments [48]. Concerning dangerous outcomes of the *infodemic* threatening human health and security, 132 member countries of the United Nations signed a cross-regional statement to combat the COVID-19 *infodemic* [49]. One of the key behavioral aims of tackling the COVID-19 *infodemic* is to increase vaccination uptakes of the world population and reach the herd immunity globally. Despite the greater need and trend of international collaboration to surveil and combat misinformation and negative languages surrounding COVID-19 vaccination, the unit of analysis in extant investigations of the impact of COVID-19 information exposure via social media has mostly been an individual within a country. Our research contributes to the literature by extending the unit of analysis to a nation and providing a global comparison. We expanded individual-level findings and showed that social media discourses are also associated with vaccination behavior at the national level. 

Consistent with previous analyses of COVID-19-related tweets, our study again confirmed a tremendous amount of attention to COVID-19 vaccines among the global public given the number of related tweets. More than 21.3 million COVID-19 vaccine tweets were posted globally within the 10-month period. Approximately 2% of COVID-19 vaccine tweets mentioned ‘death’, and the proportion was much higher in several developed countries, including Germany, Austria, Japan, and the Netherlands. It should be noted that death mentions in our data did not always refer to a death after or associated with vaccination. For instance, the term ‘death’ could be referring to how vaccines do or do not prevent COVID-19 deaths. In our analysis, death mentions were positively associated with side-effect mentions, negative sentiment, and fear/sadness/anger at the bivariate level. Furthermore, a higher proportion of death mention was observed in countries with high levels of COVID-19 morbidity, GDP per capita, literacy rate, democracy index, institutional quality, and human development index. The death mention was not a significant predictor of vaccination rates, which indicates that a more precise query strategy should be considered to capture the circulation of misinformation or rumors on vaccine-induced deaths on social media and its behavioral impacts.

More than one in 100 COVID-19 vaccine tweets mentioned ‘side-effects’ in our analysis. Side-effect mentions were also positively associated with death, negative sentiment, and fear/sadness/anger at the bivariate level. Similar with the death mentions, a greater number of side-effect mentions were observed in countries with high levels of COVID-19 morbidity, GDP per capita, literacy rate, democracy index, institutional quality, and human development index. The side-effect mentions were associated with a decreased level of vaccination rate of a country when all covariates were considered. This result indicates that, when more people in a country express concern about the side-effects of vaccines or focus on adverse events of vaccination on social media, it can discourage confidence and engagement in vaccination behaviors in the country. Social media is the double-edged sword to health communicators as it is where health misinformation spreads but offers a promising avenue to monitor and correct such misinformation with real-time corrections, crowdsourced fact-checking, and algorithmic tagging [50]. Although the health organizations and scholars should continue efforts to investigate and prevent adverse events of vaccines, as well as to provide relevant scientific information to the public, the governments and public health communicators should monitor adverse events on social media and make efforts to correct if such adverse events are incorrect or misinterpreted. Moreover, the safety and efficacy of vaccines should be communicated on social media with clear and easy information on the scarcity of adverse events to increase vaccine confidence and uptake. 

Overall, the joyous emotion appeared more frequently than fear, sadness, or anger in global tweets of COVID-19 vaccines even though some countries showed the opposite trend of showing more negative emotions. This result may reflect the world population’s excitement for a cure of the virus and possibility of ending the global pandemic. The likelihood of fear, sadness, or anger appearance as compared to joy in COVID-19 vaccine tweets was associated with a decreased level of vaccination rates. In other words, the likelihood of joy as compared to negative emotions was linked to a heightened level of vaccination rates. Health communication scholars suggested the potential of activating positive emotions in promoting vaccination by highlighting the efficacy of the vaccine in helping the return to a normal life and closer interactions with family/community after a prolonged period of social distancing [51]. Our findings support this idea at the international level. 

Our study had several limitations. First, although we found some significant correlations between variables, our analysis was limited to confirm a causal relationship between social media discourse of vaccines and vaccination due to the cross-sectional nature of our data. Second, although our macrolevel analysis may enhance ecological validity of previous studies, it would be an ecological fallacy to extrapolate country-level findings to individuals, given the difference between the unit of observation (countries) and the unit of inference (individuals). That is, the influence of Twitter discourse on individuals’ vaccine behaviors cannot directly be inferred from country-level correlations. Moreover, it is not unusual to find inflated *R*-squared values from aggregated data. Because of increased homogeneity typically found among larger-scale units (e.g., countries), correlations computed at an aggregated-level tend to be larger in general than those computed from individual-level measurements [52]. Therefore, it should be noted that some significant regression coefficients reported in our study could have been inflated, and the large *R*-squared values from our predictors could have in part been attributed to our use of aggregated data. Third, we did not explore the reasons behind why some countries showed more negative sentiment or emotions in COVID-19 vaccine tweets than others, calling for additional case studies to gain an in-depth understanding of each country’s vaccine discourse and associated factors. For instance, there can be differences in emotionally responding to COVID-19 as a crisis among countries [53]. At the same time, cross-cultural variations may exist in the public’s distrust in vaccine and other medical sciences across people with different the race/ethnicity, religions, and health literacy levels. Fourth, we did not conduct an exhaustive analysis on all COVID-19 vaccination discourses. We limited the scope of analysis to discourses related to a few adverse events of vaccination, keeping the search keywords (e.g., death, side-effects) simple and short for translation in 33 languages. Thus, our data are also limited to specific side-effects or deaths induced by COVID-19 vaccination. Due to the limitations regarding languages available for Brandwatch’s emotion analysis, we could not include several languages that are widely used in some regions that may also appear frequently in tweets (e.g., Swahili and Hausa in Africa). Another limitation is that our analysis was only based on tweets that revealed geographic locations; however, estimating geographic locations remains a challenge. Lastly, AI-driven sentiment and emotion analysis is still developing, accompanied by various limitations including Brandwatch AI’s inability to effectively detect emotions in many languages other than English. Scholars have pointed out discrepancies of sentiment and emotion classification between AI and humans [54]. Addressing these limitations can be an opportunity for future research.

Withstanding the limitations, our research contributes to the scholarship. Our algorithm-based analysis of social media conversations and macrolevel comparison of vaccination rates may enhance the ecological validity of findings from previous studies. First, instead of using questionnaire-based measures of vaccine-related concerns, attitudes, and emotions, we analyzed Twitter messages to capture real-life expressions of vaccine-related sentiment and emotions among users in the world. Second, the macrolevel comparison allowed us to analyze actual vaccination rates, rather than intention to get vaccinated, and link them to the cross-national variations in vaccine concerns, sentiment, and emotions expressed in Twitter messages. Certainly, it is questionable whether the adverse mentions, sentiment, and emotions expressed on Twitter can truly represent what the public in each country think about vaccines. That is, how closely Twitter users can represent the entire population is always questionable, and it raises concern about the validity of data from Twitter, particularly given the large variation in Twitter penetration rates across countries. Opinions of Twitter users are limited in representing the global public’s view. For instance, adult Twitter users in the US are younger and more likely to have progressive political views than the general public [55]. Furthermore, we could not include related conversations from Facebook, a social media platform with greater global market shares than Twitter, as Facebook no longer supports data access for researchers through an application programming interface (API). Despite the validity concerns, it is necessary to point out that large-scale data that can reflect and quantify cognitive and psychological responses to a universal health issue from all or most countries in the world are hardly available, and a global social media platform, such as Twitter, seems to be perhaps the only feasible source to obtain such data. The tradeoff between social media data from most countries in the world and more focused observational data from a few selected countries, i.e., the tradeoff between enhanced generalizability and higher measurement quality, seems to be an issue for current studies involving macrolevel comparisons and a task for future researchers to improve. 

## 5. Conclusions

Our research findings contribute to global efforts in communicating and promoting COVID-19 vaccination by providing a cross-national comparison of related conversations on one of the most globally used social media platforms and examining its health outcome. We found that, when social media discourse in a country focused more on side-effects or showed negative emotions when talking about COVID-19 vaccines, the country’s vaccination rate tended to be lower than others. This result considered each country’s socioeconomic and political characteristics and their COVID-19 cases and deaths. Our research supports the idea that global efforts to combat mis- and disinformation, address fear and other negative emotions, and promote positive languages surrounding COVID-19 vaccination on social media may contribute to increasing vaccination uptake worldwide.

## Figures and Tables

**Table 1:** Description of covariates.

		Total *N* = 192
	M (SD)	Min–Max
**COVID-19**		
Morbidity (per million)	59,397 (62,332)	8.60–260,309.74
Mortality (per million)	978 (1073)	3.1–6050.71
**Country Characteristics**		
GDP per capita	US$18,061 ($19,296)	661.24–116,935.6
Total population	40,867,457 (150,016,829)	10,873–1.44 billion
Population density	301 (1519)	1.98–19,347.5
Literacy rate	86% (18%)	19.10–80.90%
Democracy index ^a^	2.24 (1.08)	1–4
Institutional quality ^b^	−0.07 (0.91)	−3–1.78
Human development index ^c^	0.72 (0.15)	0.39–0.96
**Vaccination Rate ^d^**	46.99% (27.77%)	0.00–99%
Low-income countries	8.01% (9.44%)	0.00–45%
Middle-income countries	36.8% (23.6%)	1.00–84%
Upper-middle income countries	48.36% (21.37%)	8.00–90.29%
High-income countries	72.26% (12.36%)	38.83–98.99%

^a^ Democracy index scale: 1 = authoritarian regime, 2 = hybrid regime, 3 = flawed democracy, 4 = full democracy. ^b^ Composite average scores of countries on voice and accountability, political stability and absence of violence/terrorism, government effectiveness, regulatory quality, rule of law, and control of corruption (lowest score = −3.00, highest score = 3.00). ^c^ HDI scale ranges between 0 and 1 and is divided into four tiers: 1.0–0.8 = very high, 0.79–0.70 = high, 0.70–0.55 = medium, below 0.55 = low. ^d^ Percentage of total population having taken at least one dose of COVID-19 vaccine.

**Table 2:** Adverse Mentions, Negative Sentiment, and Emotions in COVID-19 Vaccine Tweets.

Vaccine Tweets	Global M (SD)	Top 10 Countries
COVID19 vaccine tweets per million users	633.19 (1941.93)	Monaco (20739.44), Canada (9302.41), Ireland (8348.63), United Kingdom (7889.12), United States (5466.66), Maldives (5082.80), Singapore (3347.87), Uruguay (2637.25), Japan (2238.79), Kuwait (2165.87)
Death mention	1.99% (2.77%)	Germany (25.60%), Austria (21.60%), Japan (12.90%), rgw Netherlands (10.68%), Liechtenstein (10.64%), Switzerland (9.43%), Suriname (4.93%), Namibia (4.90%), Swaziland (4.20%), Timor-Leste (4.08%)
Side-effects mention	1.15% (0.79%)	Burundi (4.16%), Comoros (4.00%), Germany (3.47%), Netherland (3.46%), Denmark (3.39%), Slovenia (3.24%), Macedonia (3.24%), Rep. of Congo (3.07%), Japan (2.92%), Thailand (2.95%)
Blood clots mention	0.79% (0.69%)	Equatorial Guinea (3.52%), Serbia (3.39%), Cyprus (3.89%), Swaziland (2.80%), Lesotho (2.47%), Central African Republic (2.39%), Slovenia (2.30%), Montenegro (2.22%), Mauritius (2.13%), Norway (2.08%)
Joy	*N* = 8289 (SD = 56,983)	United States (714,642), United Kingdom (228,668), India (215,465), Canada (155,620), Nigeria (28,166), Australia (25,882), Ireland (16,930), Malaysia (14,676), South Africa (13,547), Kenya (11,726)
Fear	*N* = 2315 (SD = 16,077)	United States (203,800), United Kingdom (63,378), Canada (53,693), India (42,690), Australia (14,856), Nigeria (6319), Ireland (5867), South Africa (5838), Malaysia (4457), Philippines (3552)
Sadness	*N* = 3437 (SD = 25,311)	United States (329,899), India (82,938), United Kingdom (71,629), Canada (60,401), Australia (18,144), Nigeria (11,960), South Africa (10,860), Kenya (7505), Ireland (5510), Malaysia (5008)
Anger	*N* = 1625 (SD = 12,051)	United States (151,662), United Kingdom (55,883), Canada (39,808), India (23,094), Australia (9596), South Africa (4245), Ireland (4010), Nigeria (3047), Kenya (2094), Malaysia (1607)
Likelihood of negative sentiment (vs. positive)	1.90 times (1.33)	Turkey (11.93 times), Burundi (8.73 times), Japan (6.79 times), Dem. Rep. of Congo (6.68 times), Burma (5.18 times), Togo (5.06 times), Central African Republic (4.67 times), Guatemala (4.31 times), Chad (4 times), Cape Verde (4 times)
Likelihood of fear/sadness/anger emotions (vs. joy)	0.70 times (0.33)	Namibia (1.87 times), Australia (1.65 times), Eritrea (1.63 times), Burma (1.60 times), South Africa (1.55 times), Samoa (1.52 times), Swaziland (1.50 times), Iran (1.50 times), Antigua and Barbuda (1.48 times), Iceland (1.36 times)

**Table 3:** Correlations among adverse mentions, negative sentiment, and emotions.

	Death Mention	Side-Effect Mentions	Blood Clot Mentions	Negative Sentiment	Fear/ Sadness/Anger
		*r* (*p*)	*r* (*p*)	*r* (*p*)	*r* (*p*)
Death mention		0.414 (<0.001)	0.112 (0.122)	0.235 (0.001)	0.182 (0.012)
Side-effect mentions			0.243 (<0.001)	0.338 (<0.001)	0.207 (0.004)
Blood clot mentions				−0.042 (0.568)	0.316 (<0.001)
Negative sentiment					0.306 (0.001)
**COVID-19**					
Morbidity (per million)	0.186 (<0.001)	0.224 (0.002)	0.257 (<0.001)	−0.008 (0.917)	0.080 (0.280)
Mortality (per million)	0.111 (0.137)	0.061 (0.417)	0.159 (0.033)	0.035 (0.642)	0.089 (0.236)
**Country Characteristics**					
GDP per capita	0.247 (<0.001)	0.222 (0.002)	0.168 (0.022)	0.002 (0.976)	0.231 (0.002)
Total population	0.043 (0.560)	−0.046 (0.525)	−0.072 (325)	−0.027 (713)	0.074 (0.313)
Population density	−0.003 (0.969)	0.093 (0.205)	−0.010 (0.891)	0.056 (0.444)	0.136 (0.063)
Literacy rate	0.173 (0.018)	0.132 (0.071)	0.205 (0.005)	0.039 (0.592)	0.274 (<0.001)
Democracy index	0.358 (<0.001)	0.259 (<0.001)	0.296 (<0.001)	0.008 (0.919)	0.241 (0.002)
Institutional quality	0.340 (<0.001)	0.243 (<0.001)	0.226 (0.002)	−0.029 (0.687)	0.235 (<0.001)
Human development index	0.280 (<0.001)	0.217 (0.003)	0.238 (<0.001)	−0.019 (0.796)	0.259 (<0.001)

**Table 4:** Predictors of vaccination rates.

	r (*p*)	Model I		Model II		Model III	
COVID-19		*b*	SE	*b*	SE	*b*	SE
Morbidity	0.485 (<0.001)	0.538 ***	0.000	−0.126	0.000	−0.053	0.000
Mortality	0.328 (<0.001)	−0.071	0.003	−0.035	0.002	−0.085	0.002
		Total *R*^2^ = 0.24		*R*^2^*Change* = 0.24		*R*^2^*Change* = 0.24	
**Country Characteristics**							
GDP per capita	0.642 (<0.001)			0.025	0.000	0.073	0.000
Total population	0.078 (0.283)			0.103 *	0.000	0.103 *	0.000
Population density	0.114 (0.116)			0.011	0.002	0.006	0.002 *
Literacy rate	0.671 (<0.001)			0.040	0.138	0.071	0.132
Democracy index	0.554 (<0.001)			−0.016	20.183	0.053	2.254
Institutional quality	0.690 (<0.001)			0.197	30.509	0.202	3.292
Human development index	0.812 (<0.001)			0.734 ***	260.490	0.682 ***	25.275
				*R*^2^*Change* = 0.48		*R*^2^*Change* = 0.48	
				Total *R*^2^ = 0.72			
**Vaccine Tweets**							
COVID19 vaccine tweets	0.070 (0.347)					0.052	0.003
Death mention	0.387 (0.009)					0.003	0.469
Side-effect mentions	0.003 (0.971)					−0.156 **	1.889
Blood clot mentions	0.058 (0.428)					−0.042	2.050
Negative sentiment	−0.050 (0.945)					−0.022	0.248
Fear/sadness/anger	0.144 (0.049)					−0.105 *	4.630
						*R*^2^ Change = 0.05	
						Total *R*^2^ = 0.77	

Note: *** *p* < 0.001, ** *p* < 0.01, * *p <* 0.05; *b =* standardized coefficient beta; SE = standardized error, 95%.

## Supplementary Materials

The following are available online at https://www.mdpi.com/article/10.3390/vaccines10050735/s1, Table S1: Sample Countries and Adverse Mentions, Negative Sentiment, and Emotions in COVID-19 Vaccine Tweets.
