# Supplementary material for: Adverse Mentions, Negative Sentiment, and Emotions in COVID-19 Vaccine Tweets and Their Association with Vaccination Uptake: Global Comparison of 192 Countries
Source: Vaccines (Basel). 2022 May 8;10(5):735. doi: 10.3390/vaccines10050735 (PMC9146864; doi:10.3390/vaccines10050735)
Supplement: Supplementary file 1 [file vaccines-10-00735-s001.zip › vaccines-1712342-supplementary.pdf]

Supplementary Table S1. Sample Countries and Adverse Mentions, Negative Sentiment, and Emotions in COVID-19 Vaccine Tweets

Countries	COVID19 vaccine tweets	Negative sentiment	Fear/sadness /anger	Death mention	Side effects mention	Blood clots mention
Afghanistan	2,353	.04	.24	.93	.68	.76
Albania	653	.15	.55	.99	1.38	1.84
Algeria	3,673	.03	.39	1.14	.73	.19
Andorra	141	.71	.45	2.84	.71	2.13
Angola	1,119	.09	.65	3.04	.36	.18
Antigua and Barbuda	683	.15	1.48	1.90	.44	.88
Argentina	314,804	.00	.49	1.55	.53	.13
Armenia	159	.66	.67	3.14	2.51	1.26
Australia	301,723	.00	1.65	2.42	1.36	2.05
Austria	17,042	.01	.88	21.62	2.73	1.40
Azerbaijan	655	.15	.42	1.53	1.68	.31
Bahamas	3,069	.03	.75	1.53	1.24	1.69
Bahrain	4,060	.02	.55	1.01	1.01	.54
Bangladesh	13,644	.01	.56	1.42	.79	.60
Barbados	2,528	.04	1.05	1.19	1.34	.79
Belarus	1,602	.06	.49	1.50	.56	.44
Belgium	46,449	.00	.74	3.43	1.95	1.13
Belize	1,035	.10	.65	1.45	.77	.58
Benin	1,311	.07	.18	.15	1.20	0
Bhutan	716	.14	.38	1.12	.28	.14
Bolivia	15,675	.01	.40	.80	.50	.12
Bosnia and Herzegovin a	509	.20	.78	2.36	.59	1.77
Botswana	4,501	.02	1.02	1.91	.82	.62
Brazil	1,411,131	.00	.61	2.42	.46	.07
Brunei	585	.17	1	.68	.51	.51
Bulgaria	1,631	.06	.65	1.47	1.47	1.53
Burkina Faso	875	.11	1.12	.69	1.6	.11
Burma (Myanmar)	6,479	.02	1.60	.59	.77	.34
Burundi	433	.24	.89	.92	4.16	.69
Cambodia	2,206	.05	.56	1.13	0	.54

Cameroon	6,454	.02	.64	1	.91	.84
Canada	1,457,298	.00	.99	1.68	1.06	1
Cape Verde	331	.30	.03	.60	.30	.30
Central African Republic	293	.34	.78	1.78	1.02	2.39
Chad	220	.45	.71	2.27	1.36	.45
Chile	158,213	.00	.46	1.96	.60	.19
China	17,572	.01	1.06	2.49	.95	.90
Colombia	322,557	.00	.38	1.45	.49	.13
Comoros	25	4	.00	0	4	0
Congo, Dem. Rep. of	7,189	.01	.47	1.11	1.07	.26
Congo, Rep. of	684	.15	.37	.88	3.07	.15
Costa Rica	23,536	.00	.70	1.18	.24	.24
Croatia	1,718	.06	.68	2.21	.41	1.51
Cte d'Ivoire	3,338	.03	.26	.90	1.62	.33
Cuba	28,729	.00	.63	1.38	.23	.15
Cyprus	1,248	.08	.77	.96	.96	2.88
Czech Republic	8,185	.01	.83	.96	1.34	1.11
Denmark	14,022	.01	.90	2.97	3.39	1.90
Djibouti	420	.24	.44	.95	1.43	.48
Dominica	148	.68	.39	1.35	1.35	0
Dominican Republic	50,347	.00	.56	1.07	.60	.18
East Timor (Timor-Leste)	98	1.02	.29	4.08	0	1.02
Ecuador	139,235	.00	.41	1.01	.68	.21
Egypt	11,305	.01	.73	1.14	1.04	1.20
El Salvador	46,634	.00	.54	.78	.62	.21
Equatorial Guinea	256	.39	.82	1.56	.78	3.52
Eritrea	63	1.59	1.63	1.59	0	0
Estonia	1,354	.07	.73	1.85	1.70	1.11
Ethiopia	2,759	.04	.47	1.64	.72	.83
Fiji	6,826	.01	.50	1.60	.86	.56
Finland	11,912	.01	1.00	2.03	1.56	1.28
France	581,072	.00	.71	1.96	2	.23

Gabon	1,793	.06	.14	1.00	1.23	.06
Gambia	1,689	.06	.28	1.72	.89	1.30
Georgia	745	.13	.44	.81	1.61	.54
Germany	132,112	.00	.79	25.59	3.47	.76
Ghana	43,106	.00	.47	1.61	1.81	.70
Greece	23,127	.00	.74	1.28	1.43	1.40
Grenada	433	.23	1.05	.92	1.85	2.08
Guatemala	40,414	.00	.45	1.12	.69	.16
Guinea	1,370	.07	.18	.80	1.53	.15
Guinea-Bissau	40	2.5	0	0	0	0
Guyana	1,614	.06	.65	1.61	1.12	1.67
Haiti	2,493	.04	.83	.88	1.33	.36
Honduras	22,963	.00	.39	.81	.60	.18
Hungary	2,702	.04	1.09	2.22	1.85	1.15
Iceland	1,372	.07	1.36	2.40	2.48	1.24
India	1,946,449	.00	.69	2.34	.59	.28
Indonesia	660,361	.00	.86	2.11	.91	.08
Iran	7,620	.01	1.50	3.20	.81	.59
Iraq	4,968	.02	.61	1.02	.91	.80
Ireland	126,445	.00	.91	2.06	1.17	1.06
Israel	9,530	.01	1.18	2.10	1.28	.60
Italy	91,143	.00	.64	2.11	1.77	1.19
Jamaica	22,253	.00	.71	1.63	1.18	1.63
Japan	545,845	.00	.56	12.90	2.92	.34
Jordan	4,312	.02	.63	1.16	1.02	.95
Kazakhstan	604	.17	.54	.99	.66	.17
Kenya	96,610	.00	1.02	1.87	1.31	1.21
Kiribati	58	1.72	.27	0	0	1.72
Korea, North	0	0	0	0	0	0
Korea, South	22,620	.00	.73	1.18	1.60	1.46
Kuwait	22,111	.00	.69	1.01	1.49	1.10
Kyrgyzstan	529	.19	.31	.38	.57	.19
Laos	222	.45	.22	.45	2.70	.45
Latvia	3,297	.03	.57	1.91	1.67	1.94
Lebanon	7,871	.01	1.00	1.19	2.36	1.00
Lesotho	1,054	.09	.86	3.13	.76	2.47

Liberia	571	.18	.49	1.23	1.05	.18
Libya	1,206	.08	.84	.83	1.33	1.08
Liechtenstein	235	.43	1.17	10.64	1.70	.43
Lithuania	569	.17	.52	1.05	1.75	.53
Luxembourg	1,748	.06	.90	2.17	.91	.91
Macedonia (North Macedonia)	1,174	.08	.86	2.89	3.23	1.36
Madagascar	1,015	.09	.42	1.18	1.28	.29
Malawi	3,670	.02	.81	1.14	.981	1.03
Malaysia	133,999	.00	.75	2.26	1.60	1.18
Maldives	12,066	.01	.22	.72	1.26	1.04
Mali	1,109	.09	.83	1.53	.99	.18
Malta	3,036	03	.48	1.08	1.09	1.02
Marshall Islands	1	0	0	0	0	0
Mauritania	155	.64	.90	.64	0	0
Mauritius	1,124	.08	.81	1.69	1.87	2.22
Mexico	774,267	.00	.56	.88	.59	.13
Micronesia	48	2.08	.33	4.16	0	0
Moldova	302	.33	.44	1.32	.66	.66
Monaco	2,107	.05	1.32	1.94	1.99	.71
Mongolia	4,097	.02	.44	1.00	1.22	1.22
Montenegro	349	.28	.85	1.71	.57	2.29
Morocco	3,290	.03	.52	1.09	.85	.76
Mozambique	1,342	.07	.47	1.86	.52	.15
Namibia	7,138	.01	1.86	4.90	1.43	.81
Nauru	14	7.14	.5	0	0	0
Nepal	10,819	.01	.78	1.58	.85	.57
Netherlands	81,218	.00	1.01	10.68	3.46	1.08
New Zealand	30,964	.00	1.13	1.87	1.15	.95
Nicaragua	25,417	.00	.71	.41	.22	.06
Niger	359	.28	.14	.56	.83	.28
Nigeria	218,725	.00	.76	1.43	1.43	.92
Norway	9,206	.01	1.09	2.49	1.86	2.12
Oman	5,697	.01	.66	.56	.77	.82

Pakistan	79,806	.00	.59	1.14	1.08	.76
Palau	62	1.61	1.12	0	0	1.61
Panama	48,008	.00	.76	1.37	.74	.35
Papua New Guinea	1,301	.07	.83	1.22	1.15	1.76
Paraguay	56,770	.00	.32	1.27	.47	.20
Peru	261,516	.00	.39	1.73	.27	.08
Philippines	172,958	.00	.98	1.00	1.07	.62
Poland	33,943	.00	.67	1.67	1.25	.92
Portugal	63,586	.00	.54	2.84	.46	.44
Qatar	11,981	.00	.74	.80	.91	.44
Romania	6,566	.01	.72	1.20	1.20	.53
Russia	26,543	.00	1.17	1.05	.76	.47
Rwanda	13,085	.01	.34	1.42	.61	1.01
Samoa	279	.36	1.52	2.50	1.43	.71
San Marino	99	1.01	.06	3.03	1.01	0
Saudi Arabia	139,889	.00	.68	.21	.29	.20
Senegal	6,835	.01	.30	.64	1.08	.20
Serbia	3,835	.03	1.02	2.42	1.22	3.38
Seychelles	307	.32	.38	.97	1.30	.32
Sierra Leone	986	.10	.67	1.52	.61	.60
Singapore	35,660	.00	.93	1.54	1.33	.75
Slovakia (Slovak Republic)	765	.13	.69	2.22	2.61	1.30
Slovenia	2,347	.04	.96	3.19	3.2	2.3
So Tom and Prncipe	0	0	0	0	0	0
Solomon Islands	272	.37	.41	.73	0	1.47
Somalia	2,515	.04	.38	.83	.76	1.03
South Africa	181,671	.00	1.54	3.15	1.42	1.35
South Sudan	1,132	.09	.70	2.12	1.32	.44
Spain	346,949	.00	.72	1.61	1.16	.92
Sri Lanka	11,295	.00	.69	1.08	.89	.93
St. Kitts and Nevis	378	.26	0.5	.26	.26	.26
St. Lucia	1,272	.08	.55	1.26	.08	1.26

St. Vincent and the Grenadines	2,101	.05	.73	.71	1.47	.76
Sudan	1,330	.08	.44	1.73	.61	1.12
Suriname	203	.49	.93	4.92	2.46	1.97
Swaziland (Kingdom of Eswatini)	500	0.2	1.5	4.2	1.6	2.8
Sweden	27,109	.00	.92	2.27	2.33	1.24
Switzerland	40,950	.00	.77	9.43	2.23	.53
Syria	800	.12	.72	1.25	1.25	.87
Tajikistan	79	1.26	.06	0	0	0
Tanzania	6,654	.02	.63	2.24	.96	1.36
Thailand	526,243	.00	.42	3.74	2.85	1.14
Togo	694	.14	.55	.29	1.01	0
Tonga	402	.25	.73	3.48	.99	0
Trinidad and Tobago	17,117	.01	.83	2.22	1.05	1.26
Tunisia	1,522	.07	1.01	.92	.92	.26
Turkey	159,233	.00	.65	2.53	1.55	.22
Turkmenistan	43	2.33	0.6	2.32	0	0
Tuvalu	31	3.22	1	0	0	0
Uganda	46,375	.00	.74	1.96	.94	.18
Ukraine	7,533	.01	1.19	1.04	.57	.55
United Arab Emirates	46,478	.00	.63	1.00	.64	.43
United Kingdom	1,630,589	.00	.83	2.04	1.19	1.06
United States	6,570,155	.00	.95	1.89	1.19	.58
Uruguay	46,656	.00	.47	1.91	.40	.17
Uzbekistan	262	.38	.44	1.91	1.14	1.14
Vanuatu	128	.78	.6	3.13	1.56	0
Venezuela	401,783	.00	.47	1.35	1.64	.16
Vietnam	8,814	01	.73	1.62	.77	.78
Yemen	46,480	.00	.64	1.00	.64	.43
Zambia	3,343	.03	.86	2.45	.92	.20
Zimbabwe	18,030	.01	.68	1.67	1.11	.57
